# Supplementary material for: Mechanisms governing the pioneering and redistribution capabilities of the non-classical pioneer PU.1
Source: Nat Commun. 2020 Jan 21;11:402. doi: 10.1038/s41467-019-13960-2 (PMC6972792; doi:10.1038/s41467-019-13960-2)
Supplement: Supplementary file 7 — Source data [file 41467_2019_13960_MOESM7_ESM.zip › Source_Data/FigureS2/FigureS2B_MotifScanOutput/KmeansCluster2/homerResults/motif15.similar.html]

motif15

## Information for motif15

A
C
T
G
C
T
G
A
A
C
T
G
C
G
T
A
C
G
T
A
A
T
G
C
A
C
G
T
  
Reverse Opposite:  

C
G
T
A
A
T
C
G
A
C
G
T
C
G
A
T
A
G
T
C
A
G
C
T
A
G
T
C
  

|  |  |
| --- | --- |
| p-value: | 1e-67 |
| log p-value: | -1.557e+02 |
| Information Content per bp: | 1.907 |
| Number of Target Sequences with motif | 1134.0 |
| Percentage of Target Sequences with motif | 22.58% |
| Number of Background Sequences with motif | 5266.0 |
| Percentage of Background Sequences with motif | 12.95% |
| Average Position of motif in Targets | 99.8 +/- 50.2bp |
| Average Position of motif in Background | 99.6 +/- 76.5bp |
| Strand Bias (log2 ratio + to - strand density) | 0.1 |
| Multiplicity (# of sites on avg that occur together) | 1.09 |
| Motif File: | file (matrix) reverse opposite |

### Similar de novo motifs found

|  |  |  |  |  |  |  |  |
| --- | --- | --- | --- | --- | --- | --- | --- |
| Rank | Match Score | Redundant Motif | P-value | log P-value | % of Targets | % of Background | Motif file |
| 1 | 0.754 | C G T A T C A G C G A T G A C T G T A C A G C T A G T C C T G A A G C T C T A G C T G A | 1e-39 | -90.597247 | 2.79% | 0.59% | motif file (matrix) |
| 2 | 0.768 | G T C A A T C G G T C A C G T A A T G C A G C T A T C G T C A G A G C T | 1e-33 | -76.487687 | 7.88% | 3.86% | motif file (matrix) |
